# The Inhibition of Heat Shock Protein 90 Facilitates the Degradation of Poly-Alanine Expanded Poly (A) Binding Protein Nuclear 1 *via* the Carboxyl Terminus of Heat Shock Protein 70-Interacting Protein

**DOI:** 10.1371/journal.pone.0138936

**Published:** 2015-09-28

**Authors:** Chao Shi, Xuan Huang, Bin Zhang, Dan Zhu, Huqiao Luo, Quqin Lu, Wen-Cheng Xiong, Lin Mei, Shiwen Luo

**Affiliations:** 1 Center for Experimental Medicine, The First Affiliated Hospital of Nanchang University, Nanchang, Jiangxi, China; 2 Department of Physiology, School of Basic Medicine, Tongji Medical College, Huazhong University of Science & Technology, Wuhan, Hubei, China; 3 Institute of Molecular Medicine and Genetics and Department of Neurology, Georgia Regents University, Augusta, Georgia, United States of America; 4 School of Pharmacy, Shanghai Jiao Tong University, Shanghai, China; 5 Department of Epidemiology & Biostatistics, School of Public Health, Nanchang University, Nanchang, Jiangxi, China; Emory University, UNITED STATES

## Abstract

**Background:**

Since the identification of poly-alanine expanded poly(A) binding protein nuclear 1 (PABPN1) as the genetic cause of oculopharyngeal muscular dystrophy (OPMD), considerable progress has been made in our understanding of the pathogenesis of the disease. However, the molecular mechanisms that regulate the onset and progression of the disease remain unclear.

**Results:**

In this study, we show that PABPN1 interacts with and is stabilized by heat shock protein 90 (HSP90). Treatment with the HSP90 inhibitor 17-AAG disrupted the interaction of mutant PABPN1 with HSP90 and reduced the formation of intranuclear inclusions (INIs). Furthermore, mutant PABPN1 was preferentially degraded in the presence of 17-AAG compared with wild-type PABPN1 *in vitro* and *in vivo*. The effect of 17-AAG was mediated through an increase in the interaction of PABPN1 with the carboxyl terminus of heat shock protein 70-interacting protein (CHIP). The overexpression of CHIP suppressed the aggregation of mutant PABPN1 in transfected cells.

**Conclusions:**

Our results demonstrate that the HSP90 molecular chaperone system plays a crucial role in the selective elimination of abnormal PABPN1 proteins and also suggest a potential therapeutic application of the HSP90 inhibitor 17-AAG for the treatment of OPMD.

## Introduction

Oculopharyngeal muscular dystrophy (OPMD) is a late-onset, progressive muscle disorder caused by the abnormal expansion of a poly-alanine expansion mutation in the poly(A) binding protein nuclear 1 (PABPN1) gene [1]. Patients in their fifth or sixth decades with OPMD present with initial symptoms of dysphagia and ptosis caused by the weakening of the pharyngeal and ocular muscles [2]. PABPN1 is a ubiquitously expressed protein that regulates pre-mRNA nuclear polyadenylation and influences the gene expression profiles of cells [3, 4]. In healthy populations, the (GCG)_6_ repeat in exon 1 makes up the first six of a homopolymeric stretch of 10 alanines [encoded by (GCG)_6_(GCA)_3_GCG]. In most patients with OPMD, the (GCG)_6_ repeat is expanded to (GCG)_8-13_, leading an expansion of 12–17 uninterrupted alanines located at the N-terminus of PABPN1. Several reports have shown that the enrichment of the poly-alanine expanded PABPN1 mutant leads to protein aggregation, causing intranuclear inclusions (INIs) and subsequent cell death in cultured cells and in transgenic models [5–9]. However, little is known about the molecular mechanisms relating to how mutant PABPN1 causes the disease and how the process is regulated.

Unfolded or misfolded proteins must be either refolded or eliminated by molecular chaperones or the ubiquitin proteasomal system (UPS), respectively. Previous studies have shown that the deregulation of the UPS specifically results in the accumulation and aggregation of mutant PABPN1 in animal models and patients [10]. It is believed that HSP90 serves as the chaperone protein that is required for the proper folding and structural maintenance of several proteins [11–13]. This function is achieved through ATP binding and the hydrolysis activities of HSP90 [14]. 17-(allylamino)-17-demethoxygeldanamycin (17-AAG) binds to the ATP/ADP binding pocket of HSP90, replacing the nucleotide and thereby inhibiting HSP90 activity [15]. 17-AAG stabilizes the conformation of HSP90 into an alternative one that recruits HSP70-based co-chaperone complex, which binds the misfolded client proteins and directs them to polyubiquitylation and proteasomal degradation [16, 17]. One member of the co-chaperone complex is the carboxyl terminus of heat shock protein 70-interacting protein (CHIP) [18, 19]. CHIP is a dual-function co-chaperone/ubiquitin ligase that interacts with molecular chaperones (including HSP70 and HSP90) and appears to be a master regulator of protein folding and degradation. The HSP90/HSP70/CHIP machinery has been shown to induce ubiquitination and the subsequent degradation of misfolded proteins [20]. CHIP has been connected to several neurodegenerative diseases that are characterized by protein misfolding and aggregation [21].

Although considerable effort has been made in bettering our understanding of the pathogenesis of OPMD, a direct connection between protein aggregation and OPMD remains unclear [22]. It has been shown that ubiquitin and HSP70 co-localize to INIs in the muscle of OPMD patients [5]. Furthermore, the overexpression of HSP40 and HSP70 in HeLa cells transfected with mutant PABPN1 reduced the formation of aggregates and decreased apoptosis in those cells [5, 23, 24]. These results suggest the involvement of molecular chaperone components in the modulation of mutant PABPN1 aggregation and cell survival in OPMD. However, the molecular mechanisms that regulate the stability of PABPN1 remain largely unknown.

In this study, we aimed to elucidate the molecular mechanisms by which the HSP90 chaperone system regulates PABPN1. We found that the HSP90 chaperone system plays a crucial role in the selective elimination of abnormal PABPN1.

## Materials and Methods

### Reagents and antibodies

Cycloheximide (CHX), 17-(allylamino)-17-demethoxygeldanamycin (17-AAG), and Lubrol-PX were purchased from Sigma-Aldrich (St. Louis, MO). MG-132 was obtained from Boston Biochem (Cambridge, MA). Geldanamycin was purchased from Selleck Chemicals (Houston, TX). Lipofectamine 2000 was purchased from Invitrogen (St. Louis, MO). Antibodies were purchased from Sigma (FLAG, Clone M2, F3165); ZYMED (HSP90, 37–9400); Torrey Pines Biolabs (GFP, TP401); Santa Cruz (HSP70, sc-24; anti-HA, Clone 12CA5, sc-57592); Novus (β-actin, NB600-501); Invitrogen (anti-Myc, Clone 9E10, 13–2500); Abcam (anti-CHIP, ab2917) and Dako (anti-ubiquitin, Z045801). Alexa Fluor® 594- and Alexa Fluor® 488-conjugated secondary antibodies were purchased from Invitrogen (Carlsbad, CA), and HRP-conjugated antibodies were purchased from Amersham Biosciences.

### Expression plasmids

The poly-alanine-expanded mutant and wild-type PABPN1 proteins fused to the C-terminus of EGFP (A17-PABPN1 and A10-PABPN, respectively) were generous gifts from Dr. Guy Rouleau [25]. To generate A17-PABPN1 truncated constructs, we amplified the poly-alanine fragment of PABPN1 using the Quick Change Site-Directed Mutagenesis Kit (Stratagene, La Jolla, CA) followed by cloning into the pKH3-HA vector. Mouse cDNA for ubiquitin (GenBank™ accession number AK003190) with a C-terminal HA tag were cloned into a pKH3-HA vector. Mouse cDNA for HSP90 (GenBank™ accession number NM_008302) with an N-terminal Flag-tag, HSP70 (GenBank™ accession number U73744), and mouse cDNA from CHIP (GenBank™ accession number AF129086) with a C-terminal Myc/His tag were cloned into a pcDNA3.1 plasmid vector (Invitrogen). The authenticity of all constructs was verified by DNA sequencing.

### Cell culture, transfection and immunocytochemistry

Mouse muscle C2C12 cells were propagated as myoblasts in growth medium (GM) containing DMEM supplemented with 20% FBS, 0.5% chicken embryo extract, 100 U/ml penicillin, and 100 μg/ml streptomycin at 37°C. To induce differentiation, myoblasts at 70–80% confluence were switched to a differentiation medium, which consisted of DMEM supplemented with 5% horse serum. For transfection, C2C12 myoblasts were incubated in opti-MEM serum-free medium plus plasmid DNAs and lipofectamine 2000 (Invitrogen, 11668–019) for 6 hr at 37°C before changing back to the growth medium as previously described [26]; a high transfection efficiency was routinely achieved by this protocol. In all experiments, the media were replaced daily. For immunocytochemistry, the fixed cells were incubated at 4°C overnight with primary antibodies in PBS containing 1% BSA. After washing three times with PBS, samples were incubated at room temperature for 1 hr with Alexa Fluor 488 donkey anti-mouse IgG and/or Alexa Fluor 594 goat anti-rabbit IgG. Stained cells were analyzed by confocal microscope (Zeiss, Germany).

### Immunoprecipitation and immunoblotting

Following the designed program, cells were solubilized in lysis buffer containing 0.5% Lubrol-PX, 50 mM KCl, 2 mM CaCl_2_, 20% glycerol, 50 mM Tris-HCl, and protease and phosphatase inhibitors at a pH of 7.4. Pre-cleared cell lysates were incubated with 0.5 μg of the indicated antibodies at 4°C overnight. The reaction was incubated with 50 μl of 1:1 slurry beads conjugated with protein G or A (Roche) for 3 hr at 4°C. Bound proteins were resolved by SDS-PAGE and analyzed by immunoblotting as described previously [26]. All experiments were repeated at least three times with consistent results. Bacterial GST-HSP90(233–439) fusion proteins were immobilized on glutathione sepharose 4B beads (Amersham Pharmacia), and the beads were incubated with lysates from HEK293 cells transfected with PABPN1-HA constructs. Bead-associated proteins were subjected to SDS-PAGE and immunoblotting analysis.

### Cell viability assays and aggregate counting

Cell viability was measured by the MTT assay [27]. Briefly, cells were transfected with different expression plasmids. Twelve hours post-transfection, the cells were replated into 96-well plates (5×10^3^ cells/well). Then, the cells were treated with 17-AAG (1 μM, 72 hr), and the cell viability was measured. To quantify the aggregate-containing cells, ten fields from each sample were randomly selected. The percentage of cells containing aggregates was counted, and the volume of the aggregates was measured using confocal microscope software.

### Real-time PCR

C2C12 cells were co-transfected with an HA-tagged A17-PABPN1 plasmid and GFP empty vector for the indicated times. The real-time PCR reaction was carried out as previously described [28], and the expression levels of A17-PABPN1-HA were normalized to the expression of exogenous EGFP. The primers used for real-time PCR are as follows. A17-PABPN1-HA: Forward 5’-TGC CCG AAC CAC CAA CTA CAA CAGT-3’ and Reverse 5’-CCA GCG TAA TCT GGA ACA TCG TAT GG-3’; EGFP: Forward 5’-GCA GAA GAA CGG CAT CAA GGT G-3’ and Reverse 5’-CGG ACT GGG TGC TCA GGT AGT G-3’.

### Intramuscular DNA injection and electroporation

Two month-old C57BL/6J mice were anesthetized with sodium pentobarbital, and GFP-tagged A17-PABPN1 was injected into the tibialis anterior muscles (5 μg DNA in 10 μl TE buffer). The contralateral muscles were injected with GFP-tagged A10-PABPN1 plasmid, and electric pulses were delivered using an electric pulse generator (ECM830; BTX, San Diego, CA) [26, 29]. Seven days after electroporation, A17-PABPN1 mice and A10-PABPN1 mice received intraperitoneal injections of 17-AAG (50 mg/kg) three times a week on alternate days; control mice received DMSO alone. Afterwards, the mice were sacrificed by using anesthesia at a lethal level, and the tibialis anterior muscles were fixed in cold 4% PFA for 48 hr. Individual muscle fibers were isolated and examined for INIs under a confocal microscope using GFP. Z serial images were collected and collapsed into a single image, and INI aggregates were analyzed with ZEN 2010 software (Zeiss, Germany). The protocol was approved by the committee on the ethics of animal experiments of the First Affiliated Hospital of Nanchang University (Permit Number: 2011–021) [28]. This study was carried out in strict accordance with the recommendations in the guide for the care and use of laboratory animals of the NIH.

### Statistical analysis

Data are presented as the mean ± SEM. Differences between two groups were tested using Student’s t-test and Repeated measures analysis of variance (ANOVA). ANOVA and Student-Newman-Keuls test were used to compare data across multiple groups. A two-tailed *P*<0.05 was considered to be statistically significant.

## Results

### HSP90 is a PABPN1-interacting protein

**Fig 1:**
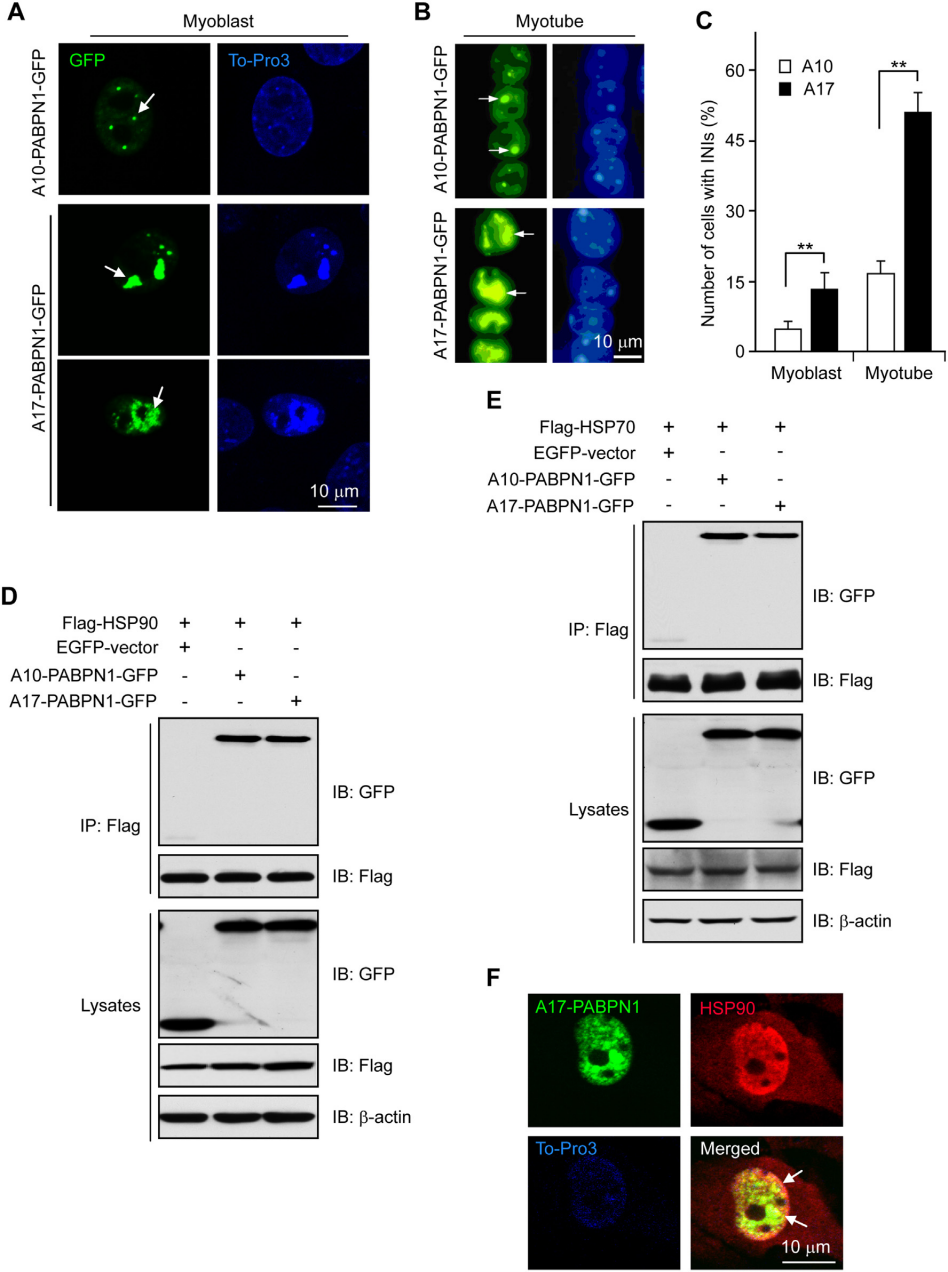
HSP90 and HSP70 interact with PABPN1. **(A-C)** A17-PABPN1 induced INIs in C2C12 cells. Mouse muscle C2C12 myoblasts were transfected with 0.5 μg GFP-tagged A10-PABPN1 or A17-PABPN1 constructs by lipofectamine 2000. Twenty-four hours post-transfection, myoblasts were fixed with 4% PFA, and the nuclei were stained with DAPI (blue). A10-PABPN1 formed small inclusions in nuclear speckles (arrows) whereas A17-PABPN1 formed large INIs **(A)**. Scale bar, 10 μm. Myoblasts were switched to differentiation medium 24 hr after transfection. Fully differentiated myotubes were fixed and stained with DAPI. INIs formed by A10-PABPN1 and A17-PABPN1 are indicated by arrows **(B)**. Cells in 10 random fields with INIs in the nucleus were scored **(C)**. Data are shown as the mean ± SEM, n = 5; *, *P* < 0.01. **(D)** Interaction of PABPN1 with HSP90. Lysates from HEK293 cells transfected with Flag-HSP90 and GFP-tagged A17-PABPN1 were immunoprecipitated with an anti-Flag antibody. Immunoprecipitates (IP) were analyzed by Western blot (IB). **(E)** Interaction of PABPN1 with HSP70. Lysates from HEK293 cells transfected with Flag-HSP70 and GFP-tagged A17-PABPN1 were immunoprecipitated with an anti-Flag antibody. **(F)** HSP90 associates with the PABPN1 aggregates. C2C12 myoblasts were transfected with A17-PABPN1 constructs, and the cells were processed for immunofluorescence staining using a HSP90 antibody 48 hr after transfection. Arrows indicate the recruitment of HSP90 to the PABPN1 aggregates. Scale bar, 10 μm.

The majority of previous experiments on PABPN1 were performed using non-muscle cells [5, 6, 30, 31]. To study the role of PABPN1 in muscle cells, we transfected C2C12 cells with A10-PABPN1 or mutant A17-PABPN1 fused to the C-terminus of EGFP (GFP-tagged A10-PABPN1 and A17-PABPN1, respectively). A17-PABPN1 is a mutant form of PABPN1 that contains 17 poly-alanines and is known to cause OPMD phenotypes in transgenic mice [32–34]. As shown in (Fig 1A–1C), A10-PABPN1 aggregates when expressed at high levels. However, the number and the size of the protein aggregates produced by A17-PABPN1 were significantly greater compared with A10-PABPN1, even at lower expression levels, which is consistent with a previous report [30].

**Fig 2:**
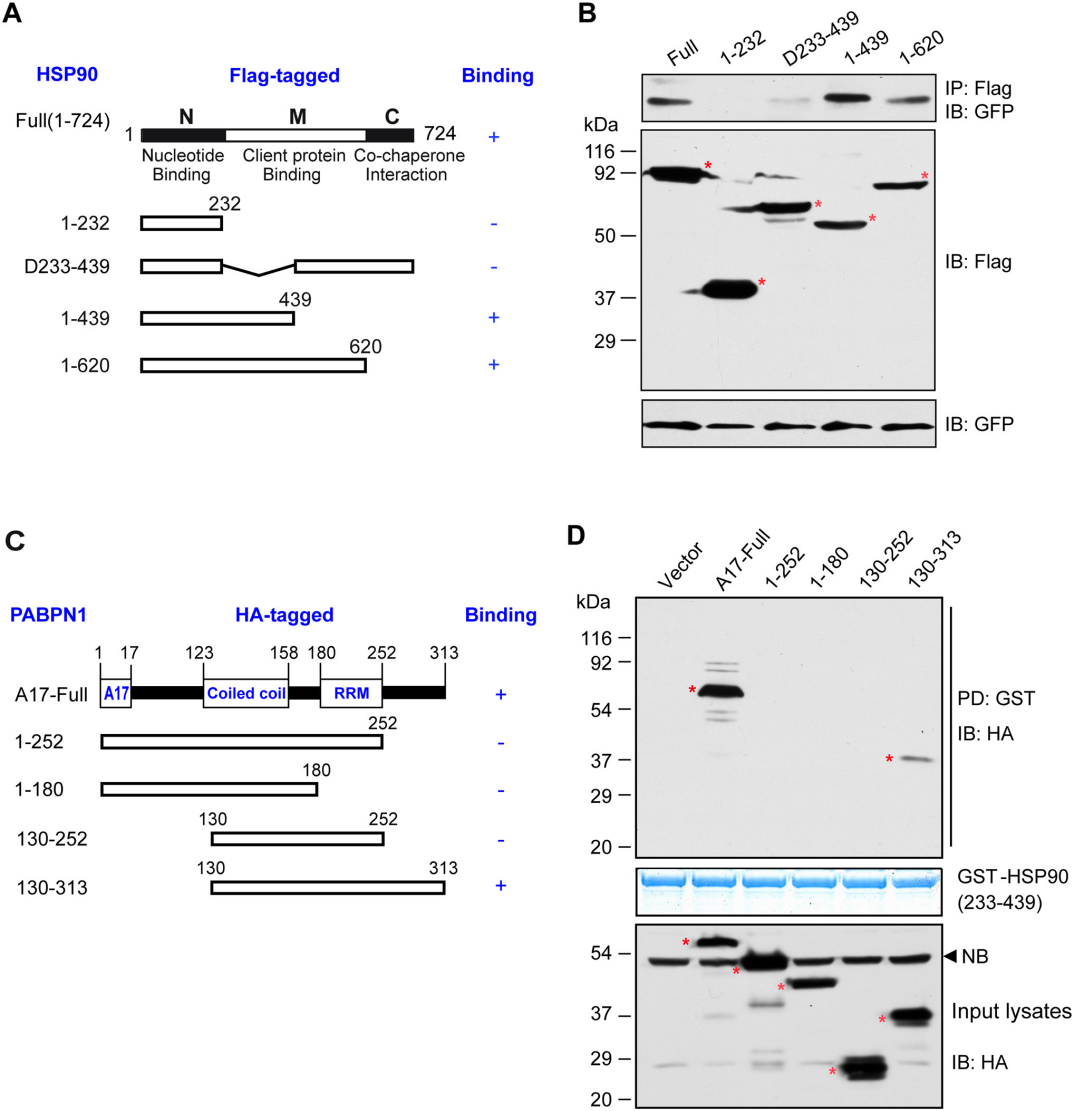
Identification of the domains responsible for the interaction between HSP90 and PABPN1. **(A)** Representation of full-length HSP90 and the structural domains used to determine the PABPN1 binding domain. **(B)** Identification of HSP90 domains involved in the PABPN1 interaction. Lysates prepared from HEK293 cells transfected with GFP-tagged A17-PABPN1 and various Flag-tagged HSP90 domain constructs were subjected to IP with an anti-Flag antibody followed by anti-GFP immunoblotting. **(C)** Representation of PABPN1 and the structural domains used to determine the HSP90 binding domain. **(D)** Identification of PABPN1 domains involved in the HSP90 interaction. Bacterial GST-HSP90(233–439) fusion proteins, immobilized on glutathione-Sepharose 4B beads, were incubated with lysates from HEK293 cells expressing various HA-tagged PABPN1 domain constructs. The precipitated proteins were subjected to anti-HA immunoblotting. A representative result from three independent experiments is shown. Asterisks highlight the various GST fusion proteins as confirmed by the comparison with the migration of molecular weight marker. IP, immunoprecipitation; PD, pull-down; NB, non-specific band.

Because INIs are hallmarks of OPMD and contain mutant PABPN1 [35], we sought to identify proteins that interact with PABPN1 with the hypothesis that they could regulate its function. A mouse muscle cDNA library (1.2 × 10^6^ clones) was screened using full length wild-type PABPN1 (A10-PABPN1) as the bait, which led to the isolation of cDNAs encoding HSP90 and HSP70. The isolated HSP90 clone contained the central region (aa 183–416) that is part of the ATPase domain and the first coiled-coil domain (Fig 2A). The HSP70 clone obtained encodes aa 401–609, containing the peptide binding domain. HSP90 and HSP70 are ubiquitously expressed ATPases that are implicated in protecting substrate proteins against aggregation and in targeting them for degradation. Given that PABPN1 nuclear aggregates are hallmarks for OPMD and are believed to be the cause of the disease, the identification of a direct interaction between heat shock proteins and PABPN1 is of considerable interest.

Although overexpression of HSP70 has been shown to suppress the aggregation of PABPN1 [5], the molecular mechanisms by which HSP70 or HSP90 would mediate such an effect require further study. To determine whether PABPN1 interacts with these heat shock proteins in mammalian cells, we co-expressed Flag-HSP90 or Flag-HSP70 with GFP–tagged A10-PABPN1 or A17-PABPN1 in HEK293 cells. The lysates of transfected cells were subjected to immunoprecipitation with an anti-Flag antibody, and the resulting precipitates were immunoblotted with antibodies against GFP or Flag. A10-PABPN1 and A17-PABPN1 were detected in the immunoprecipitates from cells co-expressing Flag-HSP90 (Fig 1D). Furthermore, A10-PABPN1 and A17-PABPN1 also co-precipitated with Flag-HSP70 (Fig 1E), confirming the results of yeast-two-hybrid screens. This interaction appeared to be specific because GFP alone was not detected in the Flag precipitates. To further study the interaction between HSP90 and PABPN1 in muscle cells, GFP-tagged A17-PABPN1 was transfected into C2C12 cells, and endogenous HSP90 was identified by immunostaining using an anti-HSP90 antibody. As shown in (Fig 1F), HSP90 co-localized with A17-PABPN1 in the nuclei. Immunostaining was negative for the transfected GFP vector alone. These data clearly support the hypothesis that HSP90 and PABPN1 physiologically interact in muscle cells.

HSP90 contains three major structural motifs: the nucleotide-binding domain in the N-terminal region that has ATPase activity; the client-binding domain in the middle region; and the C-terminal co-chaperon-interacting domain (Fig 2A). To determine the site of the interaction between HSP90 and PABPN1, we examined the interaction between PABPN1 and the different domains of HSP90. Flag-tagged truncated HSP90 (Fig 2A) and GFP-tagged A17-PABPN1 constructs were co-transfected into HEK293 cells, and the protein-protein interactions were analyzed by immunoprecipitation. As shown in (Fig 2B), the N-terminal fragment of HSP90 (aa 1–232) failed to bind to PABPN1, although full-length HSP90 bound strongly to PABPN1. Interestingly, when part of the client-binding domain in HSP90 was deleted (as in Δ233–439), the interaction between HSP90 and PABPN1 was dramatically reduced but not eliminated (Fig 2B, lane 3). The fragments containing the client-binding domain of HSP90 and its N-terminal sequence (aa 1–439 or aa 1–620) were sufficient for the interaction with full-length PABPN1 (Fig 2B, lane 4 and 5). Moreover, a GST-fusion protein containing HSP90(233–439) was able to interact with full-length PABPN1 (Fig 2D), suggesting that HSP90(233–439) is sufficient for the interaction. Similarly, our results from a yeast two-hybrid assay indicate that the client-binding domain in the middle region (aa 183–416) of HSP90 is essential for the mediation of the HSP90-PABPN1 interaction.

PABPN1 is comprised of an alanine stretch and a proline-rich region at the N-terminus, an RNA-binding domain in the central region, and a nuclear localization domain at the C-terminus [36]. We next used GST pull-down assays to examine which domains of PABPN1 were responsible for binding to HSP90 (Fig 2C and 2D). GST-HSP90(233–439) was able to pull-down the full length PABPN1 and truncated PABPN1(130–313) but did not pull-down PABPN1(1–252) or PABPN1(1–180) (Fig 2D). These results suggested that both the N-terminal region and the RNA-binding domain of PABPN1 are insufficient for the interaction with HSP90, whereas the fragment containing the C-terminal sequence (aa 252–313) was able to interact with HSP90. The deletion of this region (as in 130–252) abolished its binding to HSP90 (Fig 2D). These experiments demonstrated that the interaction occurs through the C-terminal region (aa 252–313) of the PABPN1 protein and the middle region of HSP90.

### The HSP90-specific inhibitor 17-AAG enhances the degradation of mutant A17-PABPN1

**Fig 3:**
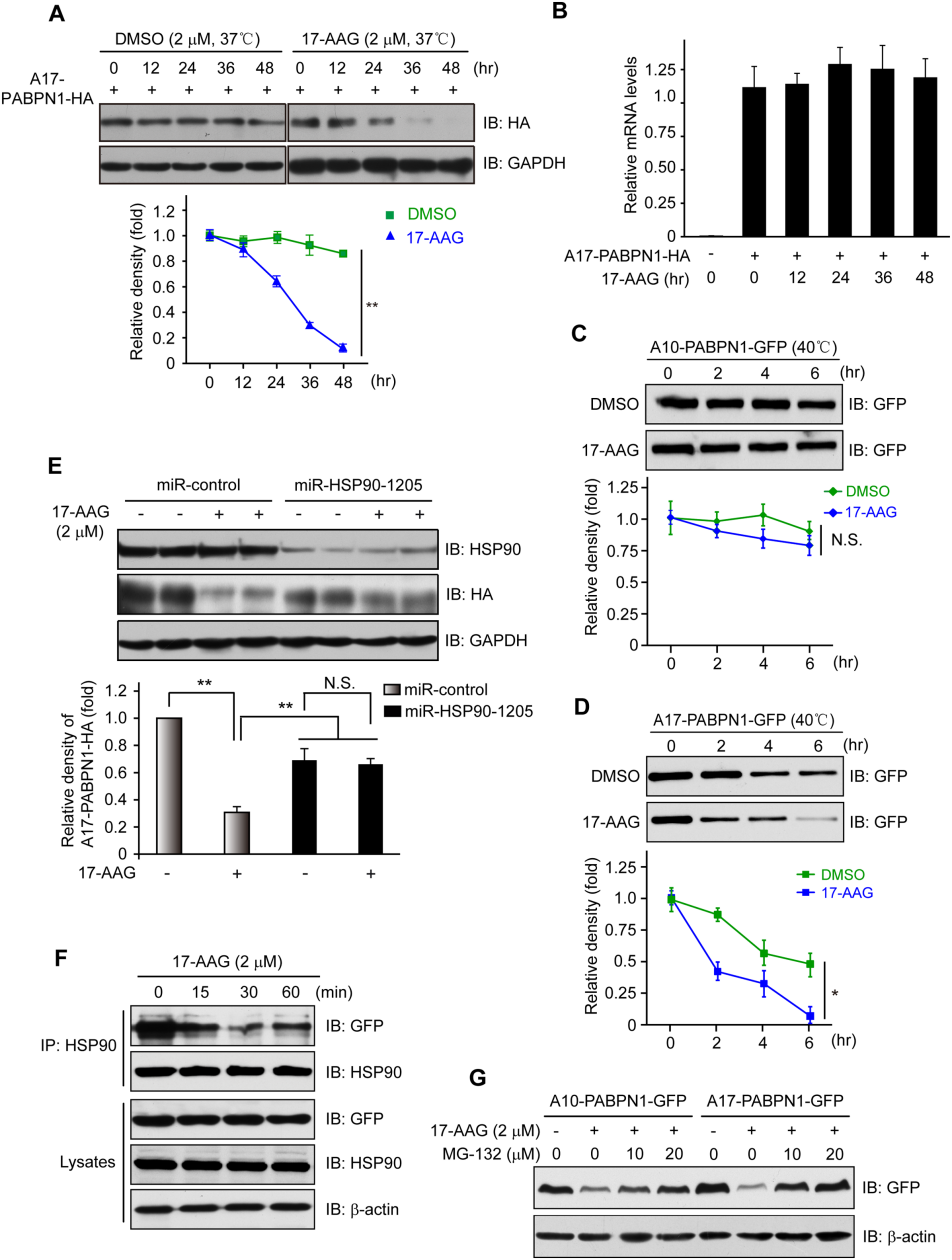
Disruption of HSP90 function induces PABPN1 degradation. **(A)** Reduction of A17-PABPN1 in 17-AAG-treated muscle cells. C2C12 myoblasts were transfected with HA-tagged A17-PABPN1 constructs. Twenty-four hours post-transfection, cells were treated with CHX (10 μg/ml) alone or together with 17-AAG (2 μM) or DMSO (2 μM) for the indicated times at 37°C. Lysates were blotted to show the expression of the proteins of interest. Band density was quantified and is shown in the line graph (bottom panel). Data are shown as the mean ± SEM (n = 5); **, *P* < 0.01. **(B)** Real-time PCR for mutant A17-PABPN1 mRNA. The mRNA levels of mutant A17-PABPN1 were similar with or without 17-AAG treatment. **(C, D)** Degradation of PABPN1 in 17-AAG treated myotubes cultured at 40°C. C2C12 myoblasts were transfected with GFP-tagged A10-PABPN1 and A17-PABPN1, and the cells were chased at 40°C in the presence of 17-AAG (2 μM) and CHX (10 μg/ml) for the indicated times 24 hr post-transfection. Lysates were blotted to show the expression of the proteins of interest. Band density was quantified and is shown in the line graphs (bottom panels). Values are mean ± SEM (n = 5). **(E)** 17-AAG reduced PABPN1 levels by 65% in cells transfected with non-silencing shRNA, whereas the suppression of HSP90 expression abrogated the 17-AAG-mediated reductions in PABPN1 expression. **(F)**. The disruption of A17-PABPN1/HSP90 complex by 17-AAG. HEK293 cells were transfected with A17-PABPN1, and the cells were treated with 2 μM 17-AAG for the indicated times 36 hr post-transfection. Cell lysates were used for immunoprecipitation and blotting with the indicated antibodies. Lysates were also blotted to show the expression of the proteins of interest (bottom panels). **(G)** The reduction of PABPN1 by 17-AAG was inhibited by MG-132. C2C12 cells were transfected with A17-PABPN1. Thirty-six hours post-transfection, the cells were treated with vehicle (DMSO) or 17-AAG (2 μM) for 8 hr. Lysates were subjected to blotting with the indicated antibodies.

HSP90 is a chaperone molecule that interacts with and stabilizes various proteins [37]. To investigate the functional consequences of the HSP90-PABPN1 interaction, we tested the half-life of A10-PABPN1 and A17-PABPN1. Both proteins appeared to be fairly stable because no measurable differences were observed within 36 hr. In the presence of 17-AAG or Geldanamycin, specific inhibitors of HSP90 [15, 38], A17-PABPN1 became less stable with a half-life of ~18 hr and ~30 hr, respectively; whereas the half-life of A10-PABPN1 was estimated to be longer than 48 hr (Fig 3A and S1 Fig). Of note, 17-AAG (2 μM, 12 hr) had no apparent effect on the viability or morphology of C2C12 myotubes. Furthermore, we found that 17-AAG had no effect on the mRNA levels of A17-PABPN1 (Fig 3B), indicating that the inhibition of HSP90 differentially regulates the stability of A10-PABPN1 and A17-PABPN1 primarily at the protein level.

To confirm the selective effect of 17-AAG on the stability of A10-PABPN1 and A17-PABPN1, we examined A17-PABPN1 and A10-PABPN1 in cells cultured at 40°C, a stress condition that is known to affect protein degradation. The degradation of PABPN1 was increased under this condition, with the half-lives being 25 hr for A10-PABPN1 and 6 hr for A17-PABPN1. In the presence of 17-AAG, the half-lives were further reduced to ~12 hr and ~2 hr for A10-PABPN1 and A17-PABPN1, respectively (Fig 3C and 3D). To investigate the molecular mechanism by which HSP90 inhibition reduces the expression of A17-PABPN1, we assessed the expression level of HSP90 in HEK293 cells that overexpressed A17-PABPN1 and were treated with 17-AAG. We found that 17-AAG reduced A17-PABPN1 expression by ~35% in HEK293 cells transfected with miRNA-control, but the knockdown of HSP90 by miRNA abrogated this 17-AAG-mediated reduction in A17-PABPN1 expression (Fig 3E), suggesting that the 17-AAG-mediated degradation of A17-PABPN1 requires a constitutive chaperone response.

To understand how 17-AAG promotes the degradation of mutant A17-PABPN1, we investigated whether 17-AAG modulates the interaction between HSP90 and PABPN1. HEK293 cells were transfected with A17-PABPN1 and then were treated with 17-AAG. Treatment with 17-AAG for one hour had no effect on the total protein levels of HSP90, β-actin, or A17-PABPN1. However, the amount of A17-PABPN1 that co-precipitated with HSP90 was reduced in 17-AAG-treated cells within 15 min of treatment (Fig 3F). These data suggest that the HSP90 inhibitor 17-AAG blocked the ability of HSP90 to stabilize A17-PABPN1, possibly through a conformational change of HSP90. Moreover, the proteasome inhibitor MG-132 blocked the 17-AAG-induced degradation of A10-PABPN1 and A17-PABPN1 (Fig 3G), suggesting that the proteasome system is required for the degradation triggered by the inhibition of HSP90. Taken together, our data demonstrated that HSP90 activity is necessary for the stability of PABPN1 and that wild-type and mutant PABPN1 could be selectively regulated by HSP90-mediated protein degradation.

### The inhibition of HSP90 decreases mutant A17-PABPN1 aggregate formation *in vitro* and *in vivo*

**Fig 4:**
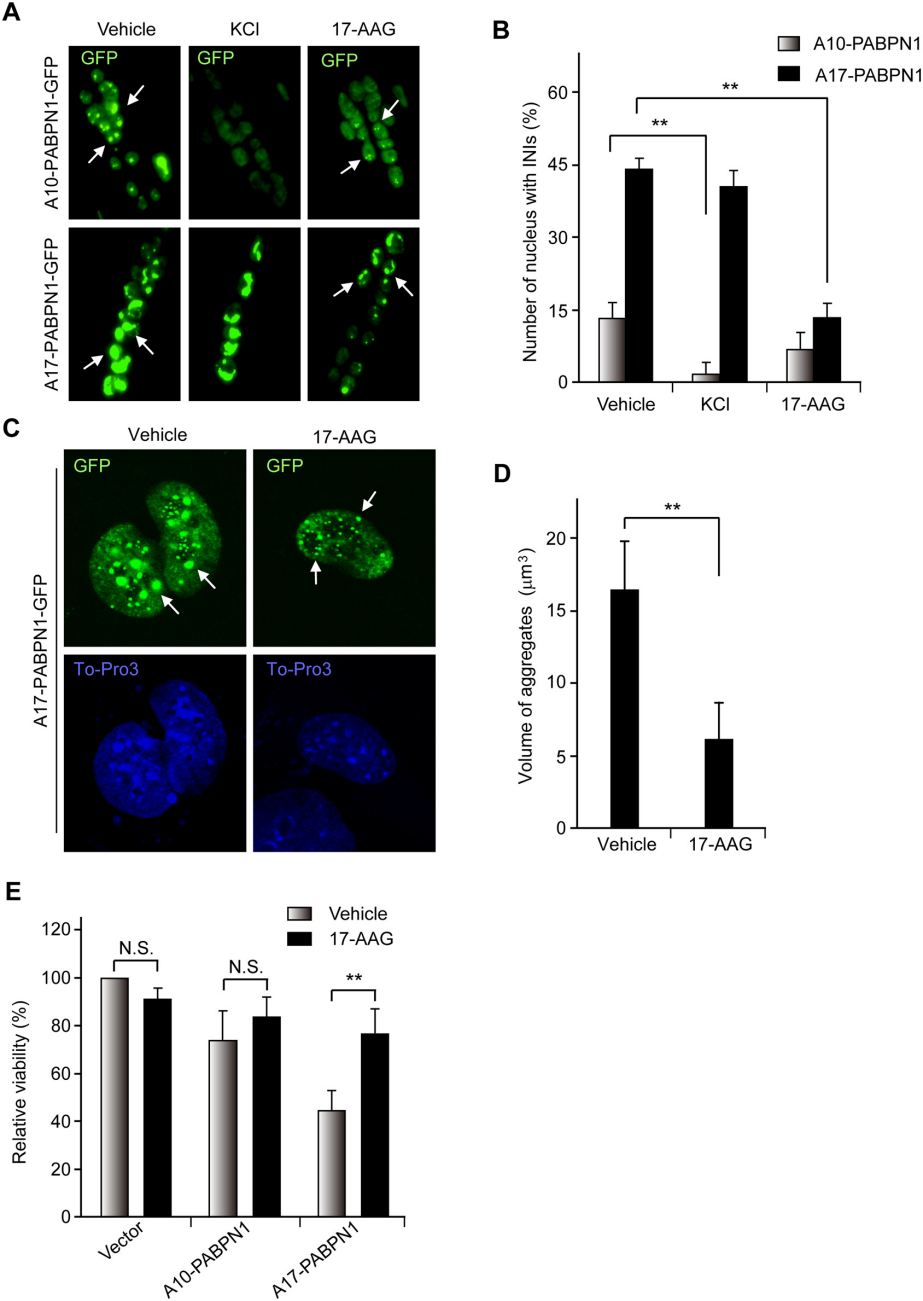
The inhibition of HSP90 reduces the aggregation and cell death caused by mutant A17-PABPN1. **(A, B)** 17-AAG decreases PABPN1-induced INIs in myotubes. C2C12 myoblasts were transfected with 0.5 mg GFP-PABPN1 constructs. Cells were switched to differentiation medium 24 hr after transfection, and fully differentiated myotubes were incubated with 1 μM 17-AAG for 12 hr; EGFP-positive myotubes were scored. In some experiments, the myotubes were treated with 1 M KCl at room temperature for 5 min to serve as a positive control. **, *P* < 0.01, n = 5. **(C, D)** The formation of A17-PABPN1 aggregates is impaired in 17-AAG treated HeLa cells. HeLa cells were transfected with GFP-tagged A17-PABPN1 constructs. Thirty-six hours post-transfection, the cells were incubated with or without 1 μM 17-AAG for 48 hr. The arrows identify cells containing INIs **(C)**. Aggregate formation was analyzed with ZEN 2010 software (~500 transfected cells in each case). The results are the means ± SEM of three independent experiments **(D)**. **, *P* < 0.01. **(E)** 17-AAG protects against mutant PABPN1 toxicity in C2C12 cells. C2C12 myoblasts were transfected with A10-PABPN1 and A17-PABPN1. Cells were harvested and replated in 96-well tissue culture plates, and the cells were treated with 500 nM 17-AAG for two days. Cell viability was measured by MTT assay. Data are mean ± SEM of three independent experiments. N.S., no significance; **, *P* < 0.01.

To explore the biological significance of the effect of 17-AAG on A17-PABPN1, we studied aggregate formation in A17-PABPN1-transfected C2C12 myotubes, a cellular model of OPMD [33]. Myotubes were transfected with A17-PABPN1 or A10-PABPN1, treated with 17-AAG and examined for INIs. PABPN1-positive INIs were observed in myotubes expressing either A10-PABPN1 or A17-PABPN1 as shown in (Fig 4A). The INIs that formed in A10-PABPN1-expressing cells were soluble in 1 M KCl whereas A17-PABPN1-positive INIs were resistant to KCl treatment (Fig 4B), which is in agreement with previous observations [32, 33]. Interestingly, the number of INIs formed by A17-PABPN1 was reduced in myotubes treated with 17-AAG, suggesting that mutant A17-PABPN1 is degraded by the 17-AAG treatment. To obtain additional evidence to support this hypothesis, we examined aggregate formation in A17-PABPN1-transfected HeLa cells in response to 17-AAG treatment alone. As shown in Fig 4C and 4D, A17-PABPN1-transfected HeLa cells that form large, visible aggregates in response to DMSO showed a reduction in the size and intensity of these aggregates in response to 17-AAG treatment (2 μM for 24 hr). To determine whether treatment with 17-AAG protects against PABPN1 toxicity, we transfected C2C12 cells with wild-type A10-PABPN1 and mutant A17-PABPN1 and studied the effects on cell viability. We found that 17-AAG (100 nM, 48 hr) significantly protected C2C12 cells against mutant A17-PABPN1-induced cell death (Fig 4E).

**Fig 5:**
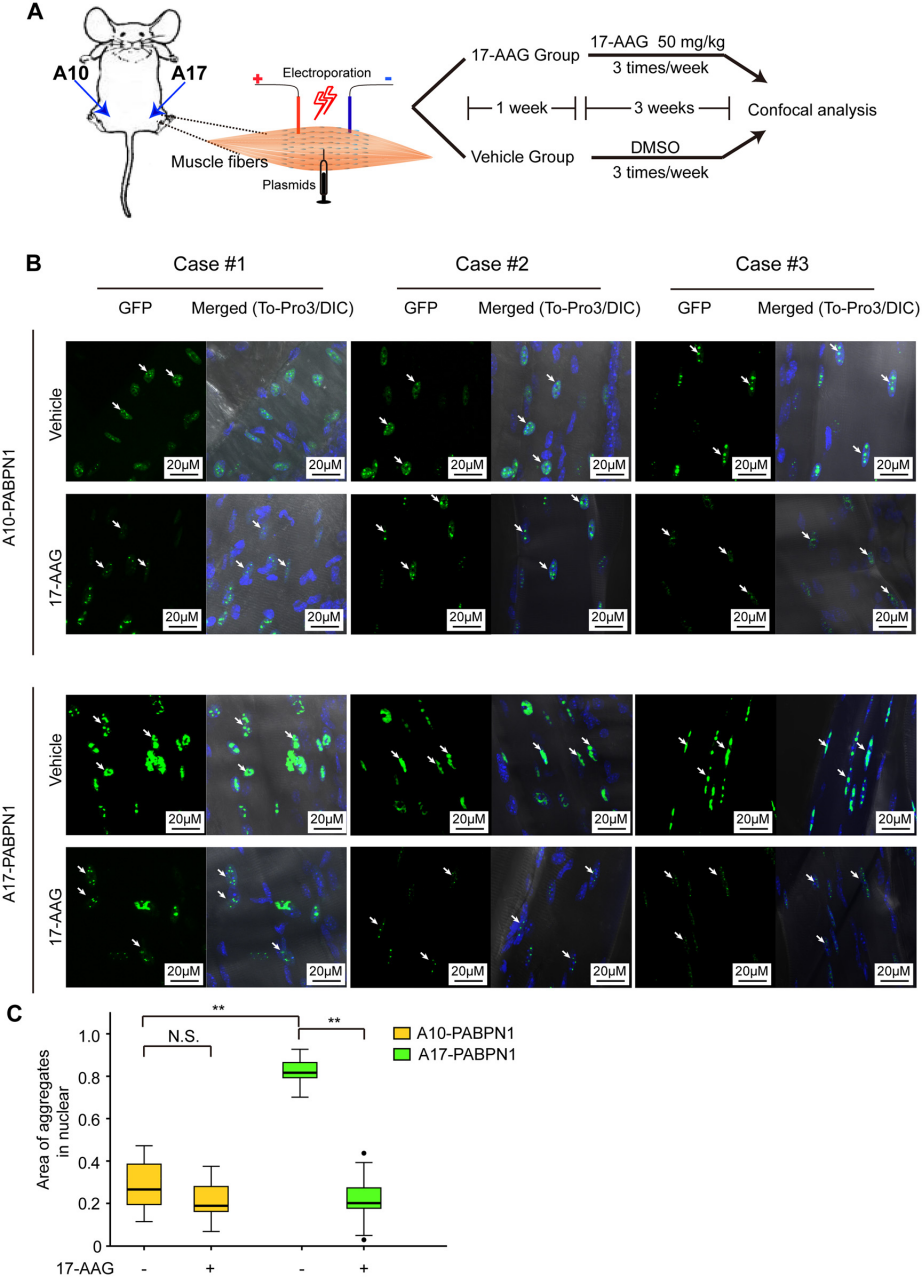
Inhibition of HSP90β enhances the degradation of mutant A17-PABPN1 aggregates *in vivo*. **(A, B)** Electroporation was performed as previously described [26, 29]. Seven days after electroporation, mice received intraperitoneal injections of 17-AAG (50 mg/kg) three times a week on alternate days for 3 weeks; control mice received DMSO alone. Individual muscle fibers were isolated and examined for INIs with GFP under a confocal microscope. Z serial images were collected and collapsed into a single image. **(C)** Quantitative analysis of the differences in the level of aggregate formation from (**B**). Data shown are mean ± SEM, n = 8; *, *P* < 0.05; **, *P* < 0.01.

As mutant A17-PABPN1 was more preferentially degraded by 17-AAG compared with wild-type A10-PABPN1 *in vivo*, we studied whether treatment with 17-AAG would have a similar effect on mutant A17-PABPN1 protein aggregates *in vivo*. A trial profile of the *in vivo* study is shown in (Fig 5A). Following treatment with 17-AAG, the amounts of aggregates in the skeletal muscle of A17-PABPN1-transfected mice were decreased by 62.5%, whereas the reduction was only ~11.5% in A10-PABPN1-transfected mice (Fig 5B and 5C). The elimination rate of the aggregates of A17-PABPN1 was markedly higher compared to A10-PABPN1 in skeletal muscle, indicating that mutant A17-PABPN1 was preferentially cleared by the inhibition of HSP90.

### Blocking HSP90 activity induces mutant A17-PABPN1 ubiquitination and degradation in a CHIP-dependent manner

**Fig 6:**
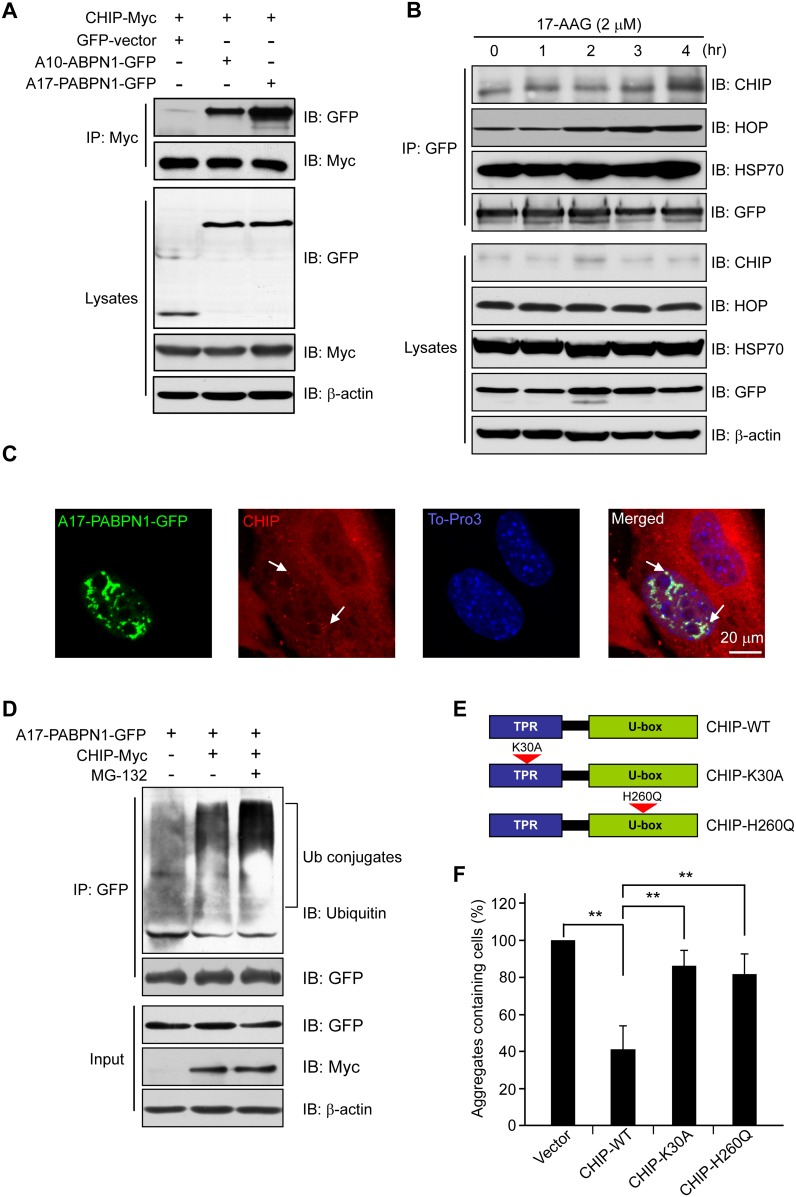
17-AAG induces PABPN1 degradation in a CHIP-dependent manner. **(A)** CHIP associates with PABPN1. HEK293 cells were co-transfected with GFP-tagged A17-PABPN1 and Myc-tagged CHIP. Lysates were immunoprecipitated with a Myc antibody 48 hr after transfection. **(B)** 17-AAG enhances the CHIP-PABPN1 interaction. A17-PABPN1-expressing myoblasts were treated with 17-AAG for the indicated times. Lysates were immunoprecipitated with anti-GFP antibody. Immunoprecipitates and total lysates were analyzed by Western blot. **(C)** The recruitment of CHIP to the mutant PABPN1 aggregates. C2C12 myoblasts were transfected with GFP-tagged A17-PABPN1 and CHIP-Myc constructs. The cells were processed for immunofluorescence staining using a Myc antibody 48 hr after transfection. Arrows indicate the recruitment of CHIP to the PABPN1 aggregates. **(D)** CHIP promotes the ubiquitination of mutant A17-PABPN1. C2C12 myoblasts were transfected and then collected and subjected to immunoprecipitation using an anti-GFP antibody. The blot was detected with an anti-ubiquitin antibody. **(E, F)** Exogenously expressed CHIP causes the degradation of mutant A17-PABPN1. C2C12 cells were co-transfected with GFP-tagged A17-PABPN1 and HA-CHIP (wild-type) or function-loss-mutant CHIP (K30A, H260Q). The number of aggregate-containing cells was counted 72 hr after transfection. **, *P* < 0.01.

CHIP is an E3 ligase that interacts with HSP90 [20]. Therefore, we investigated the possibility that CHIP ubiquitinates PABPN1 in C2C12 cells (Fig 6). First, we transiently co-transfected Myc-tagged CHIP into HEK293 cells with or without GFP-tagged A10-PABPN1 or A17-PABPN1. As shown in (Fig 6A), both A10-PABPN1 and A17-PABPN1 were found to co-immunoprecipitate with CHIP. Interestingly, mutant A17-PABPN1 showed a higher affinity for CHIP compared with wild-type A10-PABPN1, indicating a role for CHIP in the regulation of the ubiquitination and degradation of mutant A17-PABPN1. Next, we investigated the status of the HSP90 chaperone complex in A17-PABPN1-expressing C2C12 cells treated with 17-AAG. HSP70 and HOP are the essential components of the HSP90 chaperone complex [39]. Following the exposure of the transfected cells to 17-AAG, HSP70 and HOP were dramatically increased (Fig 6B), and a duration-dependent decrease in A17-PABPN1 was observed (Fig 3A). These observations indicated that HSP90 inhibition mediates a switch from the stabilizing form of the PABPN1-HSP90 chaperone complex to the proteasome-targeting form involving HOP, HSP70 and CHIP. Furthermore, we investigated the cellular localizations of CHIP and PABPN1 in C2C12 cells. C2C12 cells were transfected with GFP-tagged A17-PABPN1 and Myc-tagged CHIP. We observed co-localization of PABPN1 with CHIP (Fig 6C), suggesting a physiological interaction between PABPN1 and CHIP.

**Fig 7:**
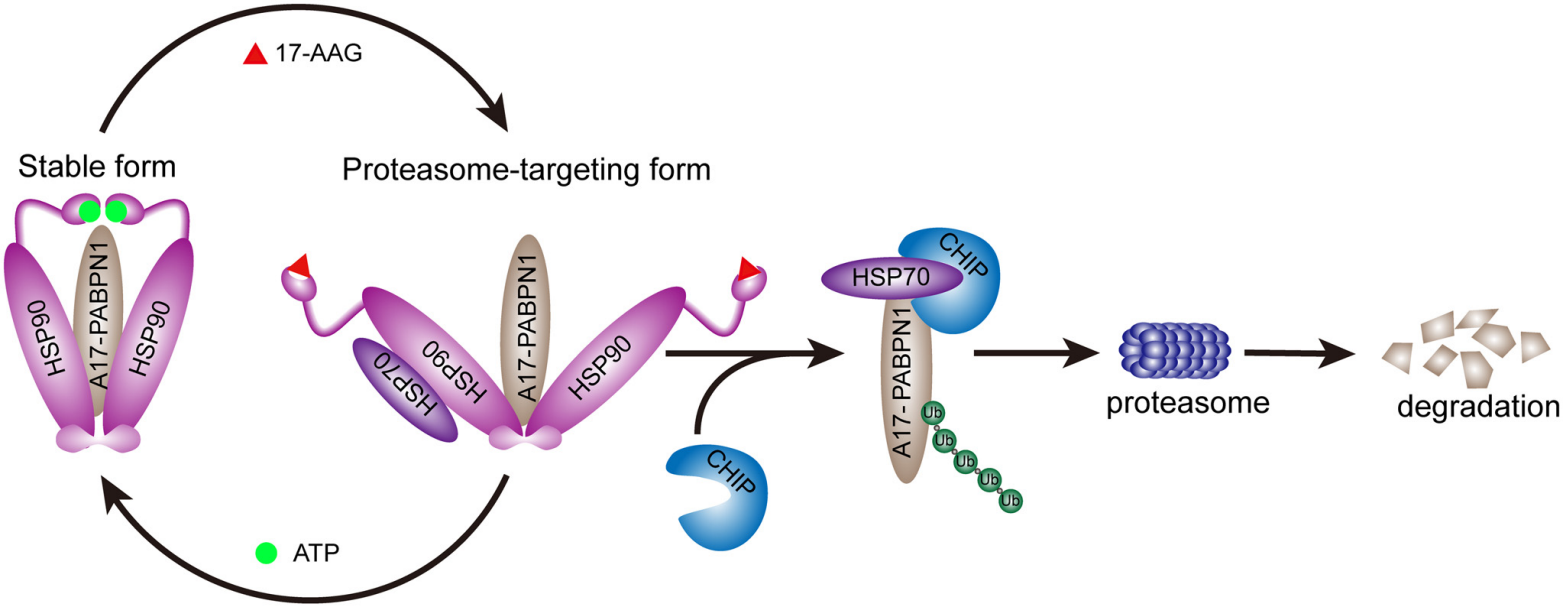
A model for the HSP90-mediated regulation of PABPN1. The blockade of HSP90 activity induces the ubiquitination and degradation of mutant A17-PABPN1 in a CHIP-dependent manner. Our findings establish that both HSP90 and CHIP play critical roles in the process of ubiquitination and degradation of mutant A17-PABPN1 proteins.

Because CHIP co-immunoprecipitates with A10-PABPN1 and A17-PABPN1 and this interaction is increased by 17-AAG (Fig 6A and 6B), we further tested the potential involvement of CHIP in the ubiquitination of the PABPN1 proteins. A17-PABPN1 was transfected into C2C12 cells with or without the CHIP construct. The cell lysates were then processed for immunoprecipitation using an anti-GFP antibody. As shown in (Fig 6D), CHIP enhanced the level of ubiquitination of A17-PABPN1. This enhancing effect was further increased by treatment with MG-132. Consistent with these results, overexpression of ubiquitin facilitated the degradation of A17-PABPN1 (S3 Fig). Because CHIP promotes the ubiquitination of mutant A17-PABPN1 proteins, we expected that its overexpression would increase the rate of degradation of mutant A17-PABPN1 proteins by proteasomes. As shown in (Fig 6E), the overexpression of CHIP reduced the aggregation of mutant A17-PABPN1. The K30A and H260Q mutants of CHIP [40, 41] lacked this suppressive effect on aggregation. Taken together, these results suggest that the blockade of HSP90 activity by 17-AAG induces A17-PABPN1 ubiquitination and degradation in a CHIP-dependent manner (Fig 7).

## Discussion

In this study, we identified HSP90 as a critical regulator of mutant PABPN1. The HSP90 inhibitor 17-AAG selectively promotes the degradation of mutant PABPN1. In addition, we found that CHIP ubiquitinates PABPN1, leading to the proteasomal degradation of PABPN1 via a HSP90 chaperone-containing complex. These findings indicate that the HSP90 chaperone system plays a crucial role in PABPN1 biology and the pathogenesis of OPMD. Thus, modulating HSP90 activity could present an effective strategy for treating mutant PABPN1-related OPMD.

HSP90, a key member of the family of ATP-driven molecular chaperones, belongs to the proteostasis network that controls protein fate [42, 43]. HSP90 functions in a multi-component complex of chaperone proteins that consists of HSP70, HOP, Cdc37, p23 and more than 20 other co-chaperones and leads to the folding, activation, and assembly of HSP90 client proteins [39, 44]. There are two main HSP90 complexes that are formed by the combination of different co-chaperones. The complex containing Cdc37 and p23 is thought to be the primary stable form, and the HSP70- and HOP-associated complex is another proteasome-targeting form that induces ubiquitin-proteasome degradation [45, 46]. 17-AAG is an ansamycin antibiotic that binds to HSP90 and inhibits the formation of the stabilized HSP90-client protein complex, resulting in the accumulation of the proteasome-targeting HSP90-based multichaperone. This form has displayed excellent efficacy in preclinical treatment of cancer and neurological disorder [17, 44, 47, 48]. In this study, we found that the HSP90 chaperone complex that is associated with HSP70, HOP and CHIP was dramatically increased following treatment with 17-AAG (Figs 3A and 6B), revealing that 17-AAG reduced the formation of the stable complex between HSP90 and PABPN1 and shifted the complex to a proteasome-targeting form, leading to the proteasomal degradation of A17-PABPN1.

The ubiquitin proteasome system is a highly conserved mechanism that is involved in the modulation of PABPN1 aggregates and cell survival in OPMD [5, 49]. One critical question is how the mutant A17-PABPN1 protein is recognized by the ubiquitination machinery and whether chaperones play a role in that process. Here, we showed that mutant A17-PABPN1 has a higher affinity for CHIP compared with wild-type A10-PABPN1 (Fig 6A). We believe it possible that the higher sensitivity of mutant A17-PABPN1 to CHIP is a critical basis for the 17-AAG-induced preferential degradation of A17-PABPN1. Previous studies have demonstrated that CHIP is a general ubiquitin ligase for misfolded proteins [50] and is responsible for the misfolding-dependent ubiquitination and degradation of mutant Copper/Zinc superoxide dismutase 1, Alpha-synuclein and Tau protein [51–54]. In this study, we observed that the overexpression of CHIP inhibits mutant A17-PABPN1 protein aggregation and cell death (Fig 6E), suggesting that mutant A17-PABPN1 proteins are degraded by proteasomes after they have been ubiquitinated by CHIP and that the removal of mutant A17-PABPN1 proteins protects cells from the toxic effects of the mutant protein.

HSP90 facilitates the folding and assembly of non-native proteins into macromolecular complexes and is also involved in aggregation prevention and protein degradation [55, 56]. Instead of protein folding, the primary function of HSP90 is thought to promote subtle changes in the conformation of client proteins, facilitating their biological functions [57]. However, NMR spectroscopy has validated that some denatured proteins (e.g., p53 and Tau) are selected and interact with HSP90 [58, 59]. In this study, we found that both A10-PABPN1 and A17-PABPN1 interact with HSP90 and HSP70 and that PABPN1 binds more to HSP90 than to HSP70. Furthermore, HSP90 interacts with PABPN1 *via* its middle region, and the C-terminal region (residues 252–313) of PABPN1 is sufficient for its interaction with HSP90.

The primary HSP90 inhibitors include radicicol and geldanamycin [60]. As a new analogue of geldanamycin, 17-AAG displays greater anti-tumor efficacy and less toxicity [48]. It is worth noting that 17-AAG has a 100-fold higher binding affinity to HSP90 in cancer cells than HSP90 derived from normal cells. One explanation for this finding is that HSP90 with a high loading of mutant client proteins has a higher level of ATPase activity compared with wild-type client protein-HSP90 complexes [61]. Furthermore, previous studies have indicated that 17-AAG accumulates and remains at high levels even 72 hr after treatment, suggesting that the inhibition of HSP90 by 17-AAG could be persistent [38]. These advantages of 17-AAG make it suitable for clinical applications, especially with regard to cancer and neurodegenerative diseases. In this study, we observed that 17-AAG promotes the degradation of mutant A17-PABPN1 more effectively than wild-type PABPN1 *in vitro* and *in vivo* (Figs 4 and 5), suggesting that HSP90 inhibitors can effectively and rapidly induce the degradation of mutant and aggregated proteins *via* the HSP90-client protein complex system.

In summary, this study provides evidence that PABPN1 interacts with HSP90 and that this interaction is necessary for the stability of PABPN1. The disruption of this interaction by 17-AAG reduces INI formation *in vitro* and *in vivo*. Furthermore, we demonstrated that the inhibition of HSP90 facilitates the degradation of mutant A17-PABPN1 *via* a CHIP-dependent ubiquitin–proteasome system. These results suggest that targeted HSP90 therapy presents a potentially effective treatment for OPMD.

## Supporting Information

S1 Fig Reduction of A17-PABPN1 in geldanamycin-treated muscle cells. C2C12 myoblasts were transfected with HA-tagged A17-PABPN1 constructs. Twenty-four hours post-transfection, cells were treated with CHX (10 μg/ml) alone or together with geldanamycin (2.5 μM) for the indicated times at 37°C. Lysates were blotted to show the expression of the proteins of interest. Band density was quantified and is shown in the line graph (right panels). Data are shown as the mean ± SEM (n = 5); **, *P* < 0.01.
(TIF)

S2 Fig HSP90 overexpression failed to increase the protein levels of A17-PABPN1 in muscle cells. The A17-PABPN1-EGFP was co-transfected with varying amounts of HSP90-Flag (0 ~ 1.6 μg DNA), or an equivalent amount of empty vector plasmid as indicated in to C2C12 cells. Forty-eight hours post-transfection, lysates were blotted to show the expression of the proteins of interest. Band density was quantified and is shown in the histograms (right panels). Data are shown as the mean ± SEM (n = 5); N.S., no significance.
(TIF)

S3 Fig Reduction of A17-PABPN1 in ubiquitin overexpressed muscle cells. The A17-PABPN1-EGFP was co-transfected with varying amounts of ubiquitin-HA (0 ~ 0.8 μg DNA), or an equivalent amount of empty vector plasmid as indicated in to C2C12 Cells. Twenty-four hours post-transfection, cells were treated with CHX (10 μg/ml) for 18 hr. Lysates were blotted to show the expression of the proteins of interest. Band density was quantified and is shown in the histograms (right panels). Data are shown as the mean ± SEM (n = 5); **, *P* < 0.01.
(TIF)
