# Supplementary figures and images for: The Inhibition of Heat Shock Protein 90 Facilitates the Degradation of Poly-Alanine Expanded Poly (A) Binding Protein Nuclear 1 via the Carboxyl Terminus of Heat Shock Protein 70-Interacting Protein
Source: PLoS One. 2015 Sep 28;10(9):e0138936. doi: 10.1371/journal.pone.0138936 (PMC4587574; doi:10.1371/journal.pone.0138936)

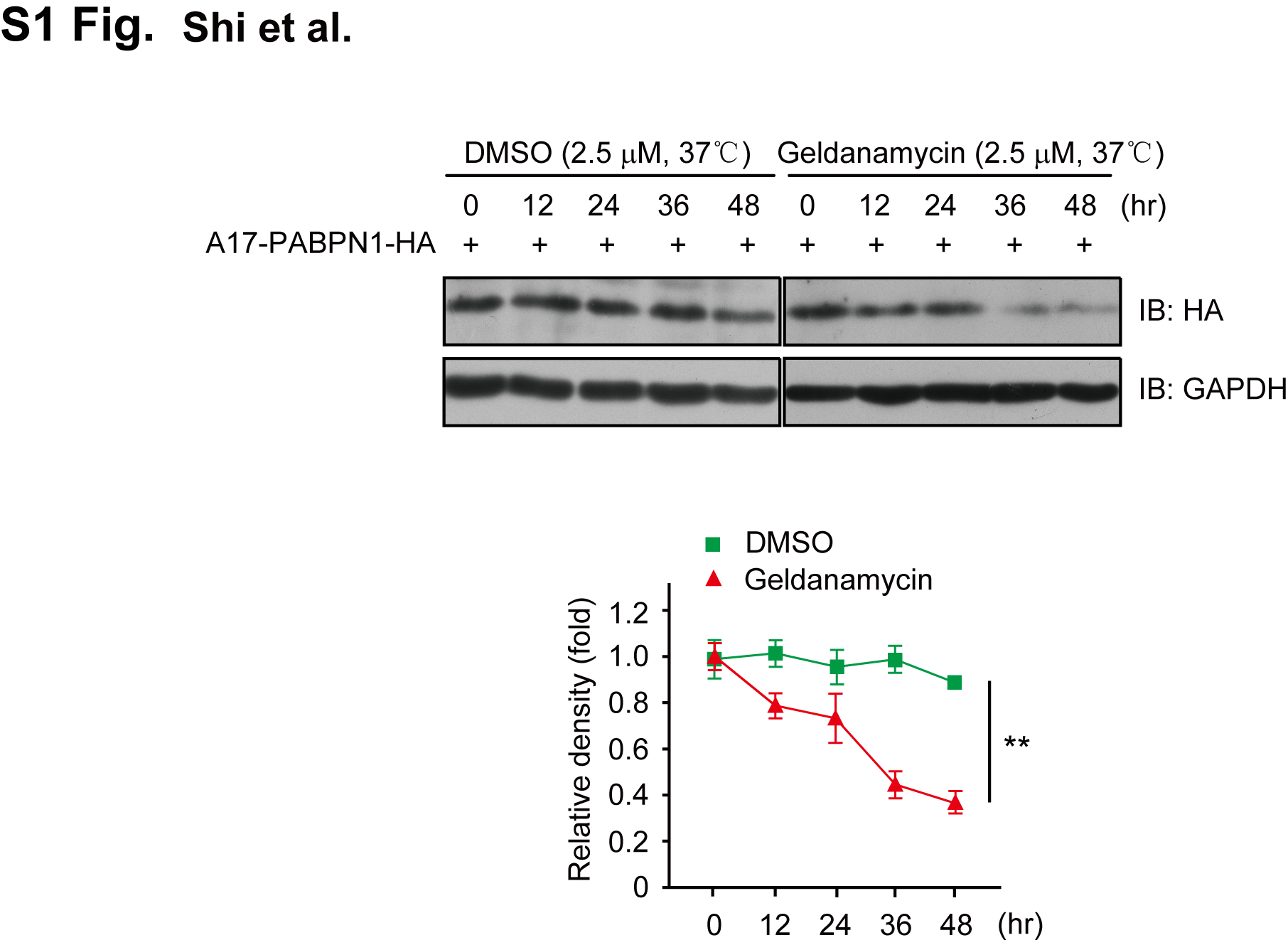

Supplement: S1 Fig — C2C12 myoblasts were transfected with HA-tagged A17-PABPN1 constructs. Twenty-four hours post-transfection, cells were treated with CHX (10 μg/ml) alone or together with geldanamycin (2.5 μM) for the indicated times at 37°C. Lysates were blotted to show the expression of the proteins of interest. Band density was quantified and is shown in the line graph (right panels). Data are shown as the mean ± SEM (n = 5); **, P < 0.01. (TIF) [file pone.0138936.s001.tif]

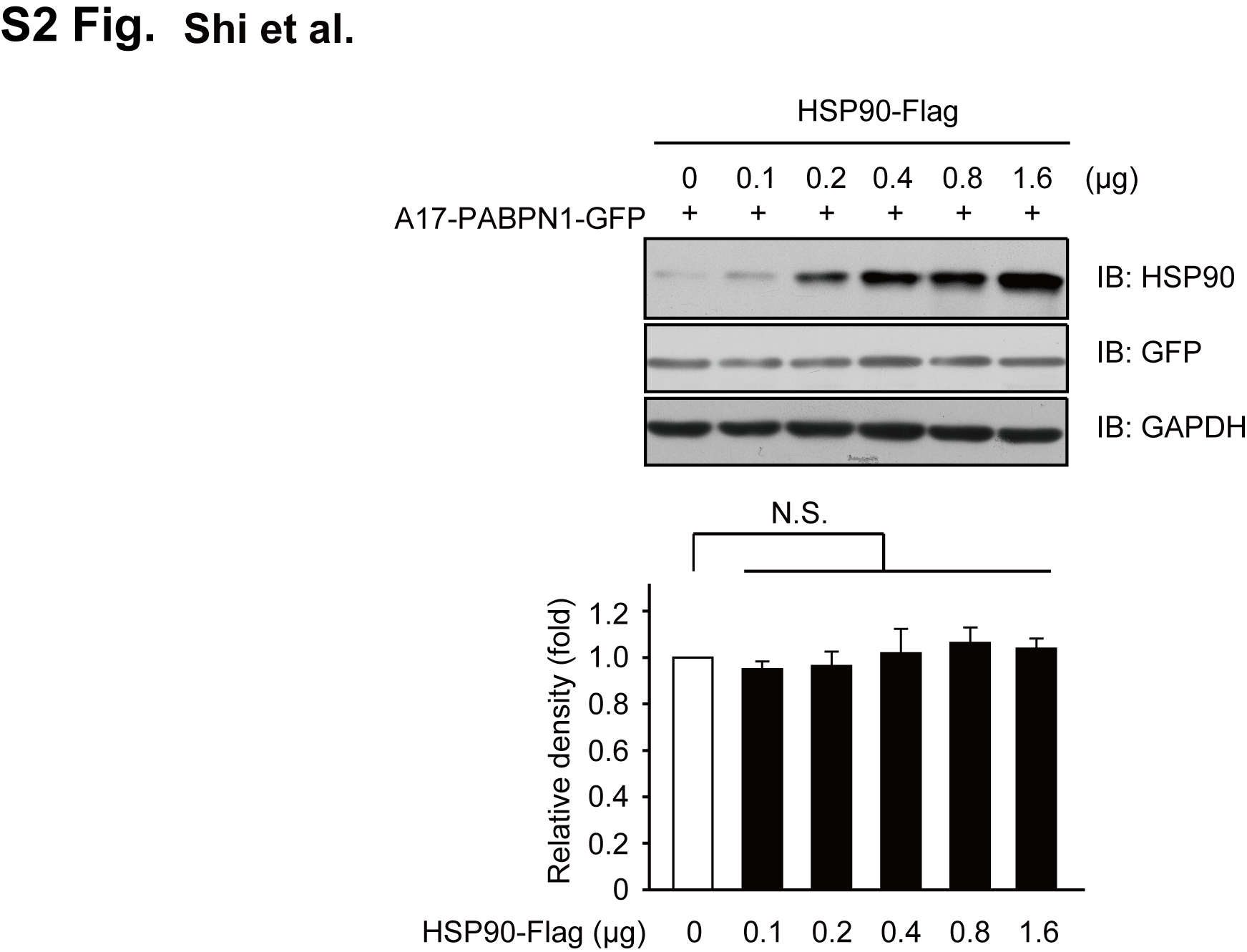

Supplement: S2 Fig — The A17-PABPN1-EGFP was co-transfected with varying amounts of HSP90-Flag (0 ~ 1.6 μg DNA), or an equivalent amount of empty vector plasmid as indicated in to C2C12 cells. Forty-eight hours post-transfection, lysates were blotted to show the expression of the proteins of interest. Band density was quantified and is shown in the histograms (right panels). Data are shown as the mean ± SEM (n = 5); N.S., no significance. (TIF) [file pone.0138936.s002.tif]

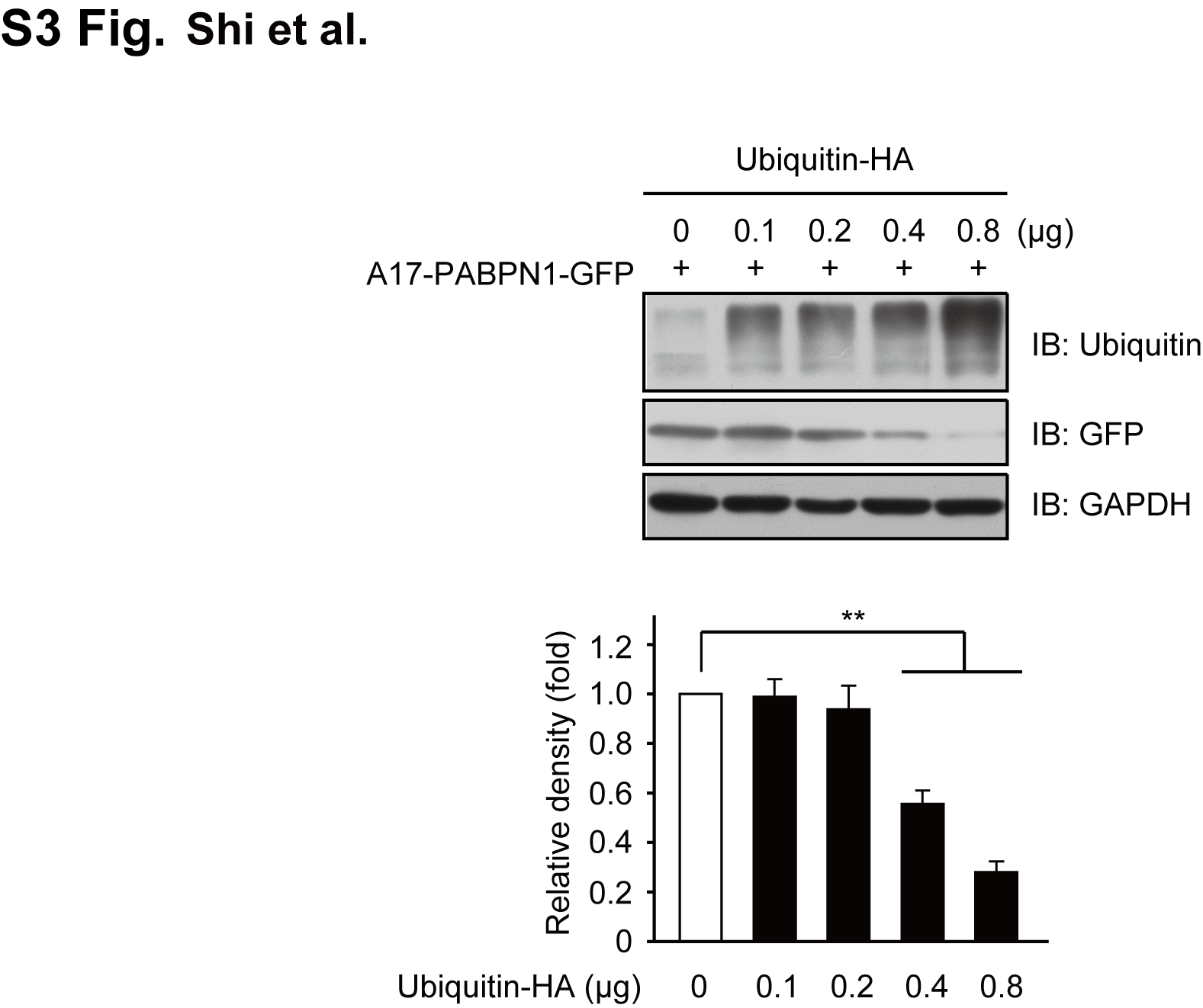

Supplement: S3 Fig — The A17-PABPN1-EGFP was co-transfected with varying amounts of ubiquitin-HA (0 ~ 0.8 μg DNA), or an equivalent amount of empty vector plasmid as indicated in to C2C12 Cells. Twenty-four hours post-transfection, cells were treated with CHX (10 μg/ml) for 18 hr. Lysates were blotted to show the expression of the proteins of interest. Band density was quantified and is shown in the histograms (right panels). Data are shown as the mean ± SEM (n = 5); **, P < 0.01. (TIF) [file pone.0138936.s003.tif]
